# A Study of Palladium Catalyzed Intra/Intermolecular Cascade Cross Coupling/Cyclizations Involving Bicyclopropylidene

**DOI:** 10.3390/molecules19056058

**Published:** 2014-05-13

**Authors:** Aydin Demircan

**Affiliations:** Department of Chemistry, Faculty of Arts &Science, Nigde University, Nigde 51240, Turkey; E-Mail: ademircan@gmail.com; Tel.: +90-532-609-27-80; Fax: +90-388-225-01-80

**Keywords:** cascade reactions, cross-coupling, palladium, cyclopropanes, electrocyclization

## Abstract

The compounds [3-(2-Bromocyclohex-2-enyloxy)prop-1-ynyl]-*tert*-butyl-dimethylsilane **3**, [4-(2-bromocyclohex-2-en-1-yloxy)but-2-yn-1-yloxy]tert-butyldimethylsilane **5** and dimethyl 2-(2-bromocyclohex-2-enyl)-2-(3-(*tert*-butyldimethylsilanyl)prop-2-ynyl)malonate **9** were prepared and subjected to palladium-catalyzed intra-intermolecular cascade cross couplings incorporating bicyclopropylidene **10** under two types of conditions. In the presence of Pd(OAc)_2_, PPh_3_ and K_2_CO_3_ in acetonitrile at 80 °C, the products were indene analogues, cross-conjugated tetraenes **11**, **12** and **13**, respectively. The corresponding spirocyclopropanated tricycle **16** in dimethylformamide at 110 °C was obtained, albeit in low yield (24%), and observed as an equimolar mixture of diastereomers, whereas **14**, **15** were not fully isolated.

## 1. Introduction

Intra- as well as intermolecular so-called domino or cascade reactions is particularly efficient in terms of increasing the molecular complexity in a minimum number of steps [1,2,3,4,5,6,7,8,9,10,11,12]. Especially, palladium-catalyzed carbon-carbon bond forming reactions that involve carbopalladations of carbon-carbon double bonds can favorably be combined in all-intra-, intra-inter- and intermolecular cascade reactions [5,6,7,8,9,10,11,12]. The so-called Mizoroki-Heck reaction, *i.e.*, the palladium-catalyzed arylation or alkenylation of alkenes has been applied in a variety of new concepts [5,6,7,8,9,10,11,12,13,14,15,16,17,18,19,20,21,22,23,24,25,26]. In view of the steadily growing number of oligocyclic compounds with cyclopropyl moieties that show interesting biological activities [27,28,29], it is noteworthy and helpful that the highly reactive building block bicyclopropylidene **10** [30,31] can participate in various palladium-catalyzed cascade reactions to furnish complex skeletons containing cyclopropyl groups [17,18,19,20] A particularly high increase in molecular complexity can be achieved with sequential reactions starting with an intramolecular carbopalladation (the first step of a Heck reaction) of a 2-bromo-1-ene-6-yne and trapping of the formed reactive vinylpalladium halide intermediate by bicyclopropylidene **10** and subsequent transformations [32]. We have been working on synthesis of fused ring systems, for indene [33,34] utilizing the intramolecular addition of alkenyl radicals to furans, for benzofuran and benzothiophene [35] towards indene also utilizing the thermal intramolecular Diels Alder cycloadduct. As this cascade reaction principle appeared to deserve further development, we embarked on a project to prepare a few more 2-bromo-1-ene-6-ynes and employ them in palladium-catalyzed cocyclizations involving bicyclopropylidene **10**.

## 2. Results and Discussion

Two major routes for the synthesis of 2-bromo-1,6-enynes as precursors for palladium-catalyzed cascade cyclizations have previously been developed [32]. Employing experimental procedures from both of these routes; three new 2-bromo-1,6-enynes with a 2-bromocyclohexene moiety for application in palladium-catalyzed cascade cyclizations with incorporation of bicyclopropylidene **10** [32] were prepared.

**Scheme 1:**
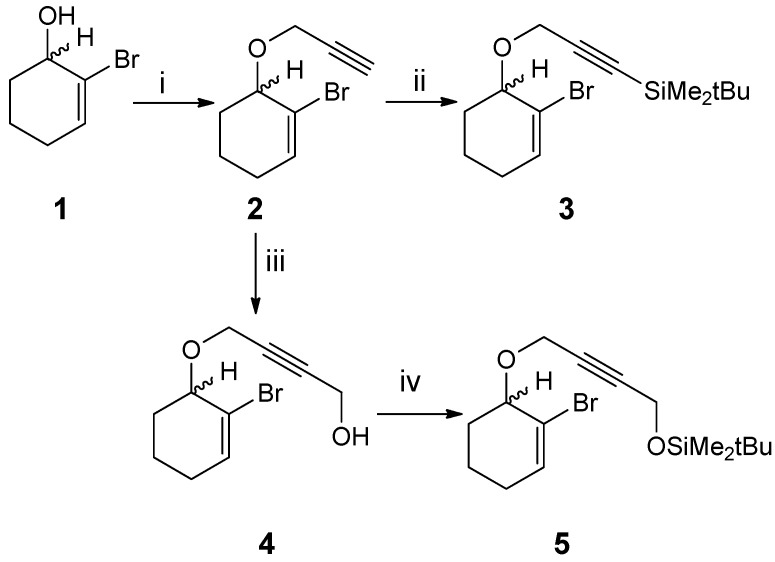
.Preparation of oxygen-containing 2-bromo-1,6-enynes.

Toward the first one, 2-bromocyclohexen-3-ol **1** [36] was deprotonated with sodium hydride and then treated with propargyl bromide to give the ether **2** (73% yield). The latter was deprotonated with *n*-butyllithium, and the resulting acetylide was trapped with paraformaldehyde to furnish the propargyl alcohol **4** (75% yield). The latter, in the presence of *N,N*-dimethylaminopyridine and imidazole, was treated with *tert*-butyldimethylsilyl chloride to yield bromoenyne **5** (75%). A second new cocyclization precursor, the silylated bromoenyne **3**, was obtained in 69% yield by trapping the acetylide from **2** with *tert*-butyldimethylsilyl chloride (Scheme 1).

*Reagents and Conditions*: i. NaH, propargyl bromide, THF, 0 °C, 73%; ii. *n*-BuLi, TBDMSCl, THF, −78 °C, 69%; iii. *n*-BuLi, (CH_2_O)_n_, THF, −78 °C, 75%; iv. DMAP, imidazole, TBDMSCl, DCM, 0 °C, 75%.

**Scheme 2:**
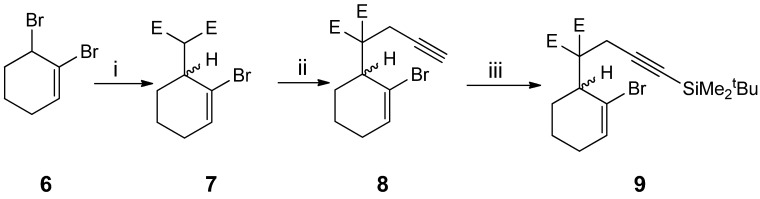
.Preparation of carbon-containing 2-bromo-1,6-enynes.

Alkylation of the sodium enolate of dimethyl malonate generated by deprotonation with sodium hydride, with 2,3-dibromocyclohexene **6** [37] provided the bromocyclohexenylbutyne **8** (82% yield), which was deprotonated at the acetylene terminus with *n*-butyllithium and the acetylide was silylated with *tert*-butyldimethylsilyl chloride to furnish **9** (58% yield) as a third cyclization precursor (Scheme 2).

*Reagents and Conditions*: i. NaH, dimethyl malonate, DME, 0 °C, 82%; ii. NaH, propargyl bromide, DME, 0 °C, 74%; iii. *n*-BuLi, TBDMSCl, THF, −78 °C, 58%, E = CO_2_Me.

**Scheme 3:**
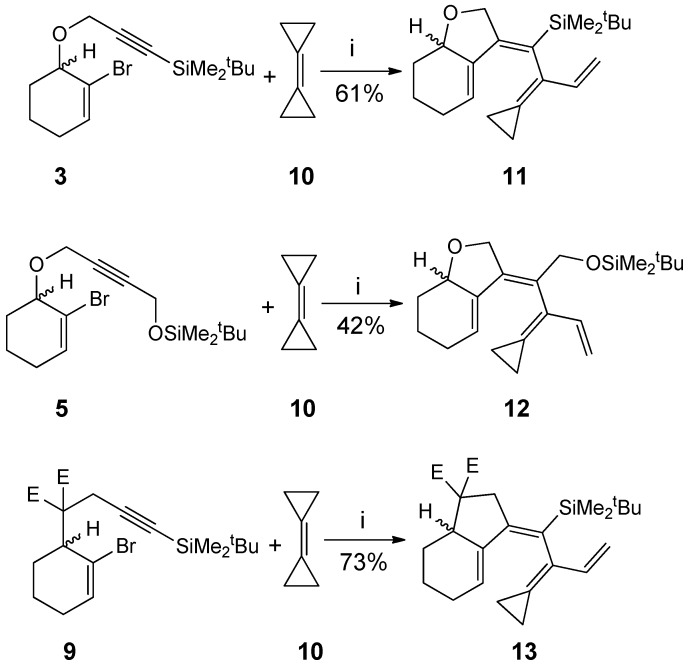
.Formation of cross-conjugated tetraenes from 2-bromoenynes and bicyclopropylidene **10**.

Under cross-coupling conditions as previously employed for palladium-catalyzed cascade co-cyclizations of 2-bromo-1-en-6-ynes and bicyclopropylidene [Pd(OAc)_2_, PPh_3_, K_2_CO_3_, MeCN, 80 °C, 5–8 h] were performed [32]. All three substrates **3**, **5** and **9** furnished cross-conjugated tetraenes with bicyclic skeletons, *i.e.*, **11**, **12** and **13**, respectively, in 61%, 42% and 73% yield (Scheme 3).

*Reagents and Conditions*: i. Pd(OAc)_2_ (10 mol %), PPh_3_ (30 mol %), K_2_CO_3_, MeCN, 80 °C, 12–14 h, E = CO_2_Me.

Mechanistically the reactions can be explained by the following sequences; initial oxidative addition of the bromo-ene onto a palladium(0) species, followed by the intramolecular carbopalladation of the triple bond to give vinyl palladium moiety, then cabopalladation of bicyclopropylidene furnish a (cyclopropylcarbynyl)palladium moiety at the intermediate which directly undergoes a cyclopropylmethyl-to-homoallyl rearrangement to bring palladium species to subsequent *syn*-β-hydride elimination stage which generates the conjugated tetraene. This corresponds to the previous observation in that the expected 6π-electrocyclizations of such initially formed tetraenes do not occur at 80 °C [32]. In the current case the tetraenes **11**, **12** and **13** did not undergo 6π-electrocyclization to the corresponding skeletons even upon heating at 100 °C for four days in a sealed tube.

**Scheme 4:**
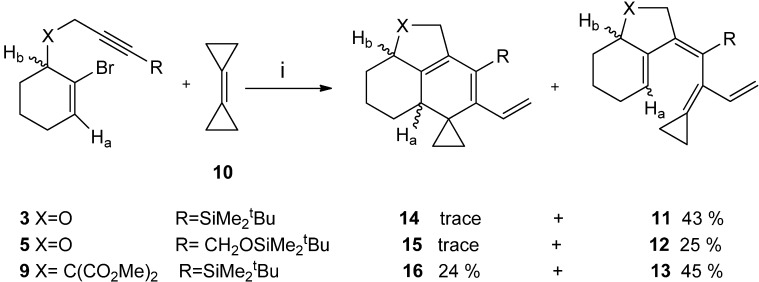
One pot domino-Heck co-cyclizations and thermal 6π –electrocyclization attemts of acyclic 2-bromo-1,6-enynes and bicyclopropylidene **10** under different contions.

The development of the yields in series **11**, **12** and **13** corresponds to that previously observed for another series with a similar substitution pattern [32]. The highest yield obtained for compound **13** obviously is due to the Thorpe-Ingold *gem*-disubstitution effect [38]. Precursors with heteroatoms in the chain consistently give lower yields than their all-carbon atoms. Presumably, heteroatoms in the chain may coordinate to the active metal catalyst and there by disturb the course or even alter mode of the reaction. When treated with palladium acetate in the presence of triphenylphosphane and potassium carbonate in *N,N*-dimethylformamide at 110 °C (instead of 80 °C), the bromoenynes **3**, **5**, **9** and bicyclopropylidene **10** underwent the desired intra-intermolecular cascade cross-coupling with subsequent 6π-electrocyclization to furnish the spirocyclopropanated tricycles for **16**, albeit in low yields (24%) as 1:1 mixtures of diastereomers regarding to NMR data, on the other hand of **14** and **15 **were not obtained and observed clearly only trace besides not cyclized **11** and **12**. (Scheme 4).

*Reagents and Conditions*: Pd(OAc)_2_ (10 mol %), PPh_3_ (30 mol%), K_2_CO_3_, DMF, 110 °C, 12−18 h.

Presumably, molecular structural features behave main role like angular torsion-difficulty, structural rigidity obstruct the 6π electrocyclization sequence or probably make reversible whereas scissor (Thorpe-Ingold) effect, with TBDMS group on compound **16** case possibly force tetraenes towards the 6π electrocyclization progress under thermal condition in a polar solvent system [33,34].

## 3. Experimental

### General Information

^1^H-NMR spectra were obtained with a Bruker 300 MHz DPX 300 spectrometer. Chemical shifts are quoted as δ values in ppm downfield from tetramethylsilane. Infrared (IR) spectra were recorded on thin films with a Perkin-Elmer 1720 spectrophotometer; Elemental analyses were carried out by TUBITAK (The Scientific and Research Council of Turkey) Marmara Research Centre on a Thermo Finnigan Flash EA 1112, (C, H, N). Electron impact (EI, 70 eV) ionization mass spectra were recorded on a Finnigan MAT 95 mass spectrometer. All reagents were used as purchased from commercial suppliers without further purification. All reactions in non-aqueous solvents were carried out using standard Schlenk techniques under a dry nitrogen atmosphere. Solvents were purified and dried according to conventional methods prior to use; tetrahydrofuran (THF), diethyl ether, dimethoxyethane (DME) were distilled from sodium/benzophenone; dichloromethane was distilled from calcium hydride. Acetonitrile and dimethylformamide were initially stirred over potassium carbonate (1 day), then over phosphorus pentoxide (1 day) and distilled (DMF was distilled under reduced pressure) onto 3Å molecular sieves. Reactions were monitored by thin layer chromatography (TLC) using pre-coated silica plates (Macherey Nagel SIL G UV_254_). Compounds were visualized using a UV lamp, alkaline potassium permanganate or acidic cerium(IV) sulfate solutions. Column chromatography was carried out on Macherey Nagel Kieselgel 60 (230−240 mesh).

*1-Bromo-6-prop-2-ynyloxy cyclohexene* (**2**): 2-Bromocyclohexen-3-ol (**1**) [38] (2.58 g, 16 mmol) in THF (10 mL) was added dropwise to the suspension of sodium hydride (60% in mineral oil, 0.75 g, 18 mmol) in THF (50 mL) kept at 0 °C. The mixture was stirred at room temperature for 30 min. Subsequently, propargyl bromide (**2**, 1.9 g, 16 mmol) was added dropwise at 0 °C. The mixture was stirred at room temperature overnight. The reaction was quenched by careful addition of brine (50 mL). The aqueous phase was extracted with diethyl ether (2 × 50 mL), the organic extracts were dried with MgSO_4_ and concentrated under reduced pressure. The residue was purified by column chromatography, eluting with pentane/diethyl ether 4:1 (*R*_f_ = 0.81), to afford **2** (2.50 g, 73%) as a pale yellow oil. IR (film): 3294 (CH), 2942 (CH), 2238 (CC), 1653 (C=C), 1083 and 1066 (C–O), 668 cm^−1^ (C-Br). ^1^H-NMR (CDCl_3_): 6.20 (t, *J =* 3.4 Hz, 1 H), 4.25 (m, 2 H), 4.0 (m, 1 H), 2.40 (t, 1 H, *J =* 2.3 Hz), 2.1–1.85 (m, 3 H), 1.8–1.4 (m, 3 H). ^13^C-NMR (CDCl_3_): 134.1, 121.9 (C_quat_), 79.9 (C_quat_), 76.3, 74.4, 57.1, 29.3, 27.7, 16.8. MS (DCI, NH_3_): *m/z* (%) = 232 (M+NH_4_^+^, 100), 186 (18), 168.1 (38). C_9_H_11_BrO (215.1), calcd. C 50.26, H 5.15; found C 50.32, H 5.10.

[3-(2-Bromocyclohex-2-enyloxy)prop-1-ynyl]-tert*-butyldimethylsilane* (**3**): *n*-Butyllithium (2.0 M in hexane, 2.5 mL, 5.1 mmol) was added dropwise to a solution of **2** (1.1 g, 5 mmol) in THF **(**25 mL) kept at −78 °C. The mixture was stirred at this temperature for 30 min, then *tert*-butyldimethylsilyl chloride (TBDMSCl, 0.74 g, 5.07 mmol) in THF (5 mL) was added, and the mixture was stirred at room temperature for 4 h. The reaction mixture was poured into water (30 mL), the mixture extracted with diethyl ether (3 × 50 mL), the organic extracts were washed with brine (50 mL), dried over MgSO_4_ and concentrated under reduced pressure. The residue was purified by column chromatography, eluting with pentane/diethyl ether 9:1 (*R*_f_ = 0.73), to afford **3** (1.15 g, 69%) as a light yellow oil. IR (film): 2952 (CH), 2857 (CH), 2173 (CC), 1645 (C=C), 1083 (C–O), 684 cm^−1^ (CBr). ^1^H-NMR (CDCl_3_): 6.2 (t, 1 H, *J =* 3.4 Hz), 4.25 (d, 2 H *J =* 4.7 Hz), 4.05 (t, 1 H, *J =* 3.2 Hz), 2.1 (m, 3 H), 1.8–1.5 (m, 3 H), 0.9 (s, 9 H), 0.0 (s, 6 H). ^13^C-NMR (CDCl_3_): 133.9, 121.2 (C_quat_), 102.4 (C_quat_), 89.7 (C_quat_), 75.9, 57.3, 29.3 (3×C), 27.8, 26.0, 16.8, 16.5, −4.7 (2×C). MS (DCI, NH_3_): *m/z* (%) = 348.3 (M+NH^+^, 100), 346 (M+NH_4_^+^, 98), 282.4 (15), 162.1 (25). C_15_H_25_BrOSi (329.3): calcd. C 54.70, H 7.65; found C 54.72, H 7.75.

*4-(2-Bromocyclohex-2-enyloxy)but-2-yn-1-ol* (**4**): *n*-Butyllithium (1.8 M in hexane, 3.9 mL, 6.98 mmol) was added dropwise to a solution of 1-bromo-6-prop-2-ynyloxy-cyclohexene (**2**, 1.5 g, 6.98 mmol) in THF **(**50 mL) kept at −78 °C. The mixture was stirred at this temperature for 30 min, then paraformaldehyde (1.03 g, 32.3 mmol) in THF (10 mL) was added, and the mixture was stirred at room temperature for 4 h. The reaction mixture was poured into water (30 mL), the mixture extracted with diethyl ether (3 × 75 mL). The organic extracts were washed with brine (75 mL), dried over MgSO_4_ and concentrated under reduced pressure. The residue was purified by column chromatography, eluting with pentane/diethyl ether 4:1 (*R*_f_ = 0.24), to afford **4** (1.33 g, 75.0%) as a light yellow oil. IR (film): 3402 (O–H), 2936 (CH), 2856 (CH), 2239 (CC), 1669 (C=C), 1024 (C–O), 668 cm^−1^ (CBr). ^1^H-NMR (CDCl_3_): 6.2 (t, *J =* 3.4 Hz, 1 H), 4.3 (m, 4 H), 4.0 (t, *J=* 3.2 Hz, 1 H), 2.1–2.0 (m, 3 H), 1.8–1.5 (m, 4 H). ^13^C-NMR (CDCl_3_): 134.1, 121.5 (C_quat_), 114.8, 84.5 (C_quat_), 82.1 (C_quat_), 57.4, 51.2, 29.3, 27.2, 16.8. MS (DCI, NH_3_): *m/z* (%) = 264.2 (M+NH_4_^+^, 98), 262.2 (M+NH_4_^+^, 100), 180.2 (60%). C_10_H_13_BrO_2_ (245.1): calcd. C 49.00, H 5.35; found C 49.08, H 5.30.

*[4-(2-Bromocyclohex-2-en-1-yloxy)but-2-yn-1-yloxy]tert-butyldimethylsilane* (5): *N,N*-Dimethyl- aminopyridine (0.18 g, 1.5 mmol) and imidazole (0.52 g, 7.5 mmol) were added to a stirred solution of 4 (1.2 g, 5 mmol) in CH2Cl2 (25 mL) kept at 0 °C, and the mixture was stirred for 15 min. *tert*-Butyldimethylsilyl chloride (1.13 g, 7.6 mmol) was added in small portions, and the mixture was stirred at room temperature for 12 h. The reaction mixture was poured into water (50 mL) and the aqueous mixture extracted with diethyl ether (3 × 50 mL). The organic extracts were dried over Na_2_SO_4_ and concentrated under reduced pressure. The residue was purified by column chromatography, eluting with pentane/diethyl ether 4:1 (*R*_f_ = 0.73), to afford **5** (1.33 g, 75%) as a colorless oil. IR (film): 2951 (CH), 2857 (CH), 2246 (CC), 1653 (C=C), 1086 and 1063 (C–O), 668 cm^−1^ (C-Br). ^1^H-NMR (CDCl_3_): 6.2 (t, *J =* 3.4 Hz, 1 H), 4.3 (m, 4 H), 4.0 (t, *J =* 3.3 Hz, 1 H), 2.1–2.0 (m, 3 H), 1.8–1.4 (m, 3 H), 0.9 (s, 9 H), 0.1 (s, 6 H). ^13^C-NMR (CDCl_3_): 133.9, 129.4, 122.2 (C_quat_), 114.8, 84.9 (C_quat_), 80.9 (C_quat_), 71.4, 56.0, 51.7, 29.3 (3×C), 18.3 (C_quat_), −5.6 (2×C). MS (DCI, NH_3_): *m/z* (%) = 378 (M+NH_4_^+^, 100), 376 (M+NH_4_^+^, 98), 262.2 (20). C_16_H_27_BrO_2_Si (359.4): calcd. C 53.47, H 7.57; found C 53.41, H 7.61.

*Dimethyl 2-(2-Bromocyclohex-2-enyl)malonate* (**7**) [35]: Dimethyl malonate (5.3 g, 40.3 mmol) was added to a suspension of sodium hydride (0.9 g, 37 mmol) in freshly distilled DME (100 mL) kept at 0 °C. The mixture was stirred for an additional 15 min after the gas evolution had ceased. 2,3-Dibromocyclohexene (**6**) [11a] (8.8 g, 36.7 mmol) was added dropwise, and the mixture was stirred at room temperature for 4 h. The reaction mixture was slowly poured into water (250 mL), the aqueous mixture extracted with diethyl ether (3 × 150 mL). The organic extracts were dried over MgSO_4_ and concentrated under reduced pressure. The residue was purified by column chromatography, eluting with pentane/diethyl ether 9:1 (*R*_f_ = 0.27), to afford **7** (8.70 g, 82%) as a colorless oil. IR (film): 2952 (CH), 2856 (CH), 1636 (C=C), 1210 and 1024 cm^−1^ (C–O). ^1^H-NMR (CDCl_3_): 6.2 (td, *J =* 3.4 Hz, *J =* 1.7 Hz, 1 H), 4.0 (d, *J* = 5.4 Hz, 1 H), 3.7 (s, 6 H), 3.2 (m, 1 H), 2.1 (m, 4 H), 1.6 (m, 2 H). ^13^C-NMR (CDCl_3_): 169.0 (C_quat_), 168.3 (C_quat_), 133, 123.3 (C_quat_), 54.0, 52.6, 52.3, 42.3, 27.5, 26.6, 19.9. MS (DCI, NH_3_): *m/z* (%) = 600.5, (2M+NH_4_^+^, 5), 310.3 (M(^81^Br)+NH_4_^+^, 100), 308 (M(^79^Br)+NH_4_^+^, 98), 244.3 (20), 211.3 (10%). C_11_H_15_BrO_4_ (291.1): calcd. C 45.38; H 5.19; found C. 45.42, H 5.07.

*Dimethyl-2-(2-Bromocyclohex-2-enyl)-2-prop-2-ynyl-malonate* (**8**): A solution of compound **7**, (4.0 g, 13.75 mmol) in DME (10 mL) was added dropwise to a suspension of sodium hydride (60% in mineral oil, 0.4 g, 16.6 mmol) in DME (50 mL) at 0 °C. The mixture was stirred at room temperature for 30 min. Subsequently, propargyl bromide (1.66 g, 14 mmol) was added dropwise at 0 °C. The reaction mixture was stirred at room temperature overnight, then it was poured into brine (75 mL) and the aqueous mixture extracted with diethyl ether (3 × 75 mL). The organic extracts were dried over MgSO_4_ and concentrated under reduced pressure. The residue was purified by column chromatography, eluting with pentane/diethyl ether 4:1 (*R*_f_ = 0.24), to afford **8** (3.34 g, 74.0%) as a colorless oil. IR (film): 2953 (CH), 2930 (CH), 2857 (CH), 2179 (CC), 1739 (C=O), 1635 (C=C), 1206 (C–O), 682 cm^−1^ (C-Br). ^1^H-NMR (CDCl_3_): 6.2 (dt, *J* = 1.7 Hz, *J* = 3.4 Hz, 1 H), 3.8 (s, 6 H, OMe), 3.6 (m, 1 H), 3.1 (dd, *J* = 2.7 Hz, *J* = 17.0 Hz, 1 H), 2.9 (dd, *J* = 2.7 Hz, *J* = 17.0 Hz, 1 H), 2.1 (m, 3 H), 1.9 (m, 2 H), 1.7–1.6 (m, 2 H). ^13^C-NMR (CDCl_3_): 169.9 (C_quat_), 169.5 (C_quat_), 135.3, 121.8 (C_quat_), 80.5 (C_quat_), 70.8, 60.4, 52.8, 52.7, 45.8, 27.5, 27.2, 23.1, 20.7. MS (DCI, NH_3_): *m/z* (%) = 348.3 (M+NH_4_^+^, 100), 346 (M+NH_4_^+^, 98), 331.2 (M+H^+^, 15), 329 (M+H^+^, 14). C_14_H_17_BrO_4_ (329.2): calcd. C 51.08, H 5.21; found C 51.16, H 5.20.

*Dimethyl-2-(2-Bromocyclohex-2-enyl)-2-[3-(t-butyldimethylsilanyl)prop-2-ynyl]malonate* (**9**) *n*-Butyl- lithium (2.5 M in hexane, 1.36 mL, 3.4 mmol) was added dropwise to a solution of **8** (1.12 g, 3.46 mmol) in THF (30 mL) kept at −78 °C. The mixture was stirred at this temperature for 30 min, then *tert*-butyldimethylsilyl chloride (0.53 g, 3.4 mmol) in THF (5 mL) was added, and the mixture was stirred at room temperature for 4 h. The reaction mixture was poured into water (75 mL), the aqueous mixture was extracted with diethyl ether (3 × 50 mL) and washed with 50 mL brine. The organic extracts were dried over MgSO_4_ and concentrated under reduced pressure. The residue was purified by column chromatography, eluting with pentane/diethyl ether 4:1 (*R*_f_ = 0.44), to afford **9** (0.90 g, 58.0%) as a light yellow oil. IR (film): 2952 (CH), 2858 (CH), 2179 (CC), 1736 (C=O), 1679 (CC), 1093 (C–O), 659 cm^−1^ (CBr). ^1^H-NMR (CDCl_3_): 6.2 (t, *J =* 3.4 Hz, 1 H), 3.8 (s, 6 H), 3.6 (m, 1H), 3.2 (d, *J* =17 Hz, 1 H), 2.8 (d, *J =* 17 Hz, 1 H), 2.0 (m, 4 H), 1.6 (m, 2 H), 0.9 (s, 9 H), 0.0 (s, 6 H). ^13^C-NMR (CDCl_3_): 170.1 (C_quat_), 169.8 (C_quat_), 135.1, 122.0 (C_quat_), 103.4 (C_quat_), 85.7 (C_quat_), 52.7 (C_quat_), 52.6, 45.9, 27.5, 27.3 (3xC), 25.9, 24.5, 22.3, 20.5, 16.5, –4.6 (2xC). MS (DCI, NH_3_): *m/z* (%) = 904.7 (2M+NH_4_^+^, 100), 460.4 (M+NH_4_^+^, 10), 445.3 (M (^81^Br) + H^+^, 98), 443.3 (M(^79^Br)+H^+^, 96), 363.4 (90). C_20_H_31_BrO_4_Si (443.4): calcd. C 54.17, H 7.05; found C 54.44, H 7.06.

*GP1: General Procedure for the Palladium-Catalyzed Cocyclization of 2-Bromo-1,6-enynes and Bicyclopropylidene* (**10**) *to Yield Tetraenes*: Palladium acetate (11.2 mg, 49.9 µmol, 10 mol %), triphenylphosphane (39.3 mg, 150 µmol, 30 mol %) and potassium carbonate (138 mg, 998 µmol) were suspended in anhydrous acetonitrile (5 mL) in a screw-cap Pyrex bottle. The respective bromoenyne (0.5 mmol) was added and argon gas was bubbled through the mixture for 5 min. Then bicyclopropylidene **10** (80 mg, 1 mmol) was added, the bottle was tightly closed, and the mixture heated in a preheated oil bath at 80 °C for the given period of time. After cooling down to room temperature, the reaction mixture was taken up in diethyl ether (10 mL), the mixture filtered through a pad of Celite (5 cm) and the solvent evaporated *in* *vacuo* to leave about 1 mL. The crude product was purified by flash column chromatography on silica gel, eluting with pentane/diethyl ether.

*GP2: General Procedure for the Palladium-Catalyzed Cocyclization of 2-Bromo-1,6-enynes and Bicyclopropylidene* (**10**) *with Subsequent 6π-Electrocyclization*: Palladium acetate (11.2 mg, 49.9 µmol, 10 mol %), triphenylphosphane (39.3 mg, 150 µmol, 30 mol %) and potassium carbonate (138 mg, 998 µmol) were suspended in anhydrous DMF (5 mL) in a screw-cap Pyrex bottle. The respective bromoenyne (0.5 mmol) was added and argon gas was bubbled through the mixture for 5 min. Then bicyclopropylidene **10** (80 mg, 1 mmol) was added, the bottle was tightly closed, and the mixture was heated at 110 °C in a preheated oil bath for the given period of time. After cooling down to room temperature, the reaction mixture was directly added onto a pre-packed column of silica gel and the column eluted with pentane/diethyl ether.

*tert-Butyl-[2-cyclopropylidene-1-(5,6,7,7a-tetrahydrobenzofuran-3-ylidene)but-3-enyl]dimethylsilane* (**11**): Treatment of compound **3**, (165 mg, 0.5 mmol) and bicyclopropylidene (**10**, 80 mg, 1 mmol) according to GP1 for 12 h gave **11**. Purification by column chromatography, eluting with pentane/diethyl ether 9:1 (*R*_f_ = 0.43), gave a compound **11** (100 mg, 61%) as a yellow oil. IR (film): 3055 (CH), 2954 (CH), 2931 (CH), 2858 (CH), 1653 (C=C), 1036 cm^−1^ (C–O). ^1^H-NMR (CDCl_3_): 6.5 (dd, *J* = 17.8 Hz, *J* = 10.5 Hz 1 H), 5.8 (t, *J* = 3.5 Hz, 1 H), 5.0 (dd, *J* = 1.9 Hz, *J* = 11.5 Hz 1 H), 4.9 (dd, *J* = 1.9 Hz, *J* = 11.5 Hz 1 H), 4.6 (dd, 1 H), 4.4 (dd, *J* = 2.4 Hz, *J* = 12.9 Hz, 1 H), 4.0 (m, 1 H), 2.15 (m, 2 H), 2.8 (m,1 H), 1.4–1.0 (m, 6 H, Cpr + 2 H), 0.9 (s, 9 H), 0.0 (s, 6 H). ^13^C-NMR (CDCl_3_): 148.2 (C_quat_), 138.6 (C_quat_), 136.4, 132.7 (C_quat_), 128.3 (C_quat_), 123.6 (C_quat_), 123.3, 113.6, 78.2, 72.6, 28.4, 27.8 (3xC), 26.3, 20.0, 18.7 (C_quat_), 4.21, 2.76, −3.4 (2xC). MS (DCI, NH_3_): *m/z* (%) = 674.7 (2M+NH_4_^+^, 15), 346.4 (M+NH_4_+, 25), 329.4 (M+H^+^, 60), 251.3 (100). C_21_H_32_OSi (328.6): calcd. C 76.77, H 9.82; found 76.61, H 9.86.

*tert-Butyl-[3-cyclopropylidene-2-(5,6,7,7a-tetrahydrobenzofuran-3-ylidene)pent-4-enyloxy]dimethylsilane* **(12)**: Treatment of compound **5**, (179 mg, 0.5 mmol) and bicyclopropylidene (**10**, 80 mg, 1 mmol) according to GP1 for 12 h gave **12**. Purification by column chromatography, eluting with a pentane/diethyl ether 10:1 (*R*_f_ = 0.51) gave the title compound **12** (64 mg, 42%) as a yellow oil. IR (Film): 2952 (CH), 2856 (CH), 1686 (C=C), 1018 cm^−1^ (C–O). ^1^H-NMR (CDCl_3_): 6.5 (dd, *J* = 17.8 Hz *J* = 10.5 Hz, 1 H), 6.1 (m, 1 H), 5.0 (m, 2 H), 4.4 (m, 2 H), 4.0 (m, 2 H), 2.2 (m, 2 H), 2.0–1.8 (m, 2 H), 1.6–1.4 (m, 4 H, Cpr), 1.2 (m, 2 H), 0.9 (s, 9 H), 0.0 (s, 6 H). ^13^C-NMR (CDCl_3_): 140 (C_quat_), 138.7 (C_quat_), 136.6 (C_quat_), 128.1, 124.5, 122.2 (C_quat_), 115.3, 70.1 (C_quat_), 66.4, 46.0, 30.1, 29.7, 27.2 (3xC), 22.1, 20.8, 18.7 (C_quat_), 10.1, 8.3, −4.0 (2xC). MS (DCI, NH_3_): *m/z* (%) = 298.4 (100), 227.2 (25), 149.2 (55). C_22_H_34_O_2_Si (326.6): calcd. C 73.69, H 9.56; found C 73.73, H 9.58.

*Dimethyl 3-[1-(tert-Butyldimethylsilyl)-2-cyclopropylidenebut-3-en-1-ylidene]-2,3,5,6,7,7a-hexahydro-1H-indene-1,1-dicarboxylate* (**13**): Treatment of compound **9** (222 mg, 0.5 mmol) and bicyclopropylidene (**10**, 80 mg, 1 mmol) according to GP1 for 14 h, after purification by column chromatography, eluting with pentane/diethyl ether 9:1 (*R*_f_ = 0.36) gave 13 (162 mg, 73%) as a yellow oil. IR (film): 3054 (CH), 2953 (CH), 2857 (CH), 2739, 2708, 1734 (C=O), 1653 (C=C), 1093 cm^−1^ (C–O). ^1^H-NMR (CDCl_3_): 6.5 (dd, *J* = 17.8 Hz, *J* = 10.5 Hz 1 H), 6.0 (t, *J* = 3.4 Hz, 1 H), 5.10 (dd, *J* = 1.9 Hz, *J* = 11.5 Hz, 1 H), 5.0 (dd, *J* = 1.9 Hz, *J* = 11.5 Hz, 1H ), 3.8 (s, 6 H, OCH_3_), 3.2 (t, *J* = 6.2 Hz, 1 H), 3.0 (m, 1 H), 2.8 (d, *J* = 16.2 Hz, 1 H), 2.0 (m, 2H), 1.6 (m, 2 H), 1.4 (m, 2 H), 1.2–1.0 (m, 4 H, Cpr), 0.9 (s, 9 H), 0.0 (s, 6 H). ^13^C-NMR (CDCl_3_): 172.2 (C_quat_), 170.7 (C_quat_), 147.6 (C_quat_), 138.7 (C_quat_), 137.8 (C_quat_), 133.4 (C_quat_), 131.3 (C_quat_), 125.5, 124.9 (C_quat_), 113, 6, 60.8, 52.5, 51.8, 47.2, 42.4, 28.1 (3xC), 26.4, 25.9, 21.8, 18.9 (C_quat_), 4.09, −3.1 (2xC), 2.5. MS (DCI, NH_3_): *m/z* (%) = 905 (2M+NH_4_^+^, 18), 460.5 (M+NH_4_^+^, 100), 443.5 (M+H^+^, 60%), 347.4 (68), 327.2 (18), 300.2 (20). C_26_H_38_O_4_Si (442.7): calcd. C 70.55, H 8.65; found C 70.48, H 8.72.

*Dimethyl 3-(tert-Butyldimethylsilyl)-4-ethenyl-6,7,8,8a-tetrahydro-1H-spiro[acenaphthylene-5,1' cyclopropane]-1,1-(2H,5aH)dicarboxylate* (**16**): Treatment of compound **9** (222 mg, 0,5 mmol) and bicyclopropylidene **10** (80 mg, 1 mmol) according to GP2 for 12 h, after purification by column chromatography, eluting with pentane/diethyl ether 10:1 (R_f_ = 0.32) gave 16 (53 mg, 24%) as pale yellow oil. IR (film): 2952 (CH), 2856 (CH), 1734 (C=O), 1654 (C=C), 1251, 1055 cm^−1^ (C–O). ^1^H-NMR (CDCl_3_): 6.1 (dd, J = 17.8 Hz, J = 11.5 Hz, 1 H), 5.8 (dd, J = 2.0 Hz, J = 11.5 Hz, 1 H), 4.8 (dd, J = 2.0 Hz, J = 11.5 Hz, 1 H), 3.8 (m, 1 H), 3.7 (s, 6 H), 3.0 (d, J = 2.6 Hz, 1 H), 2.82 (d, J = 2.6 Hz, 1 H), 2.1 (brm, 1 H), 1.8 (m, 2 H), 1.6 (m, 2 H), 1.2 (m, 2 H), 0.9 (s, 9 H), 0.6–0.3 (m, 4 H, Cpr), 0.0 (s, 6 H). ^13^C-NMR (CDCl_3_): 172.7 (C_quat_), 170.7 (C_quat_), 152.**0** (C_quat_), 139.2 (C_quat_), 136.0, 128.2 (C_quat_), 125.8 (C_quat_), 119.1, 61.8 (C_quat_), 52.6, 52.2, 45.5, 43.2, 34.1, 30.5, 27.8, 27.5 (3xC), 26.8, 21.4 (C_quat_), 19.2 (C_quat_), 16.3, 14.5, ‒ 4.0 (2xC). MS (DCI, NH_3_): m/z (%) = 460.5 (M+NH_4_^+^, 82), 459.5 (M-H+NH_4_^+^, 100), 443.5 (M+H^+^, 60), 365.4 (85), 177.2 (85). C_26_H_38_O_4_Si (442.7): calcd. C 70.55, H 8.65; found C 70.54, H 8.65.

## 4. Conclusions

The new 2-bromo-2-en-6-ynes **3**, **5** and **9** each containing a bromocyclohexene initiator, like the previously investigated completely acyclic analogues, [32] undergo palladium-catalyzed intra-intermolecular cascade cross coupling reactions in the presence of bicyclopropylidene **10** to give indene analogues, conjugated tetraenes, **11**, **12**, **13** (in MeCN at 80 °C). The reaction, under different conditions (in dimethylformamide at 110 °C) the desired spirocyclopropanated tricycle **16** was obtained albeit in low yield (24%) and observed as an equimolar mixture of diastereomers, whereas others **14**, **15** were not fully isolated. Although the yield of spirocyclopropanated tricycle **16** was rather disappointing, the concept of the 6π-electrocyclizations proved to be feasible on less strained compounds. Additionally, an alternative palladium-mediated process rather than its free radical version we studied before [33,34,35] give indene skeletons with higher yield.

## References

[1] Tietze L.F., Beifuss U. (1993). Sequential Transformations in Organic-Chemistry - A Synthetic Strategy with a Future. Angew. Chem. Int. Ed. Engl..

[2] Tietze L.F., Lieb M. (1998). Domino reactions for library synthesis of small molecules in combinatorial chemistry. Curr. Opin. Chem. Biol..

[3] Tietze L.F., Modi A. (2000). Multicomponent domino reactions for the synthesis of biologically active natural products and drugs. Med. Res. Rev..

[4] Tietze L.F. (1996). Domino reactions in organic synthesis. Chem. Rev..

[5] De Meijere A., Meyer F.E. (1994). Fine Feathers Make Fine Birds - The Heck Reaction in Modern Garb. Angew. Chem. Int. Ed. Engl..

[6] De Meijere A., Bräse S., Murakhashi S.İ., Davies S.G. (1999). Transition Metal Catalyzed Reactions.

[7] De Meijere A., Bräse S. (1999). Palladium in action: Domino coupling and allylic substitution reactions for the efficient construction of complex organic molecules. J. Organomet. Chem..

[8] Bräse S., de Meijere A., de Meijere A., Diederich F. (2004). Metal-Catalyzed Cross-Coupling Reactions.

[9] De Meijere A., von Zezschwitz P., Bräse S. (2005). The virtue of palladium-catalyzed domino reactions - Diverse oligocyclizations of acyclic 2-bromoenynes and 2-bromoenediynes. Acc. Chem. Res..

[10] Negishi E., Copéret C., Ma S., Liou S.-Y., Liu F. (1996). Cyclic carbopalladation. A versatile synthetic methodology for the construction of cyclic organic compounds. Chem. Rev..

[11] De Meijere A., von Zezschwitz P., Nüske H., Stulgies B. (2002). New cascade and multiple cross-coupling reactions for the efficient construction of complex molecules. J. Organomet. Chem..

[12] De Meijere A., Schelper M., Knoke M., Yücel B., Sünnemann H.W., Schenrich R.P., Arve L. (2003). Palladium-catalyzed cross-coupling reactions and electrocyclizations - efficient combinations for new cascade reactions. J. Organomet. Chem..

[13] Henniges H., Meyer F.E., Schick U., Funke F., Parsons P.J., de Meijere A. (1996). Palladium-Catalysed Oligocyclisations of 2-Bromododeca-1,11-diene-6-ynes. Tetrahedron.

[14] Schweizer S., Tokan W.M., Parsons P.J., de Meijere A. (2010). Palladium-catalyzed oligocyclizations of 2-bromoalka-1,(n+m+1)-dien-(n+1)-ynes - Influence of tether lengths and substituents on the outcome of the reaction (Part II). Eur. J. Org. Chem..

[15] Tokan W.M., Meyer F.E., Schweizer S., Parsons P.J., de Meijere A. (2008). Palladium-Catalyzed Oligocyclizations of 2-Bromoalk-1-ene-(n+1),(m plus n+1)-diynes - Influence of Tether Lengths and Substituents on the Outcome of the Reaction (Part I). Eur. J. Org. Chem..

[16] Tokan W.M., Schweizer S., Thies C., Meyer F.E., Parsons P.J., de Meijere A. (2009). Palladium-Catalyzed Cascade Oligocyclizations Involving Competing Elementary Steps Such as Thermal [1,5]-Acyl Shifts. Helv. Chim. Acta.

[17] Nüske H., Bräse S., Kozhushkov S.I., Noltemeyer M., Es-Sayed M., de Meijere A. (2002). New highly efficient three-component domino Heck-Diels-Alder reaction with bicyclopropylidene: Rapid access to spiro[2.5]oct-4-ene derivatives. Chem. Eur. J..

[18] Bräse S., Wertal (nee Nüske) H., Frank D., Vidovic D., de Meijere A. (2005). Intramolecular Heck couplings and cycloisomerizations of bromodienes and enynes with 1',1'-disubstituted methylenecyclopropane terminators: Efficient syntheses of [3] dendralenes. Eur. J. Org. Chem..

[19] Yücel B., Arve L., de Meijere A. (2005). A two-step four-component queuing cascade involving a Heck coupling, pi-allylpalladium trapping and Diels-Alder reaction. Tetrahedron.

[20] Yücel B., Valentic N., Noltemeyer M., de Meijere A. Cyclopropyl building blocks in organic synthesis, part 143. A two-step, three-component queuing cascade leading to dihydrobenzoxepine and dihydrobenzazepine derivatives. Eur. J. Org. Chem..

[21] Seo J.H., Liu P., Weinreb S.M. (2010). Evolution of a Strategy for Total Synthesis of the Marine Fungal Alkaloid (+/−)-Communesin F. J. Org. Chem..

[22] Priebbenow D.L., Henderson L.C., Pfeffer F.M., Stewart S.G. (2010). Domino Heck-Aza-Michael Reactions: Efficient Access to 1-Substituted Tetrahydro-beta-carbolines. J. Org. Chem..

[23] Vlaar T., Ruijter E., Orru R.V.A. (2011). Recent Advances in Palladium-Catalyzed Cascade Cyclizations. Adv. Synth. Catal..

[24] Charpenay M., Boudhar A., Blond G., Suffert J. (2012). An Expeditious and atom-economical synthesis of a new generation of substituted [4.6.4.6]fenestradienes. Angew. Chem. Int. Ed..

[25] Young D.W. (2010). An upfront investment. Nat. Chem. Biol..

[26] Leibeling M.,  Milde B., Kratzert D., Stalke D., Werz D.B. (2011). Intermolecular Twofold Carbopalladation/Cyclization Sequence to Access Chromans and Isochromans from Carbohydrates. Chem. Eur. J..

[27] Salaun J., Baird M.S. (1995). Biologically-Active Cyclopropanes and Cyclopropenes. Curr. Med. Chem..

[28] Salaun J. (2000). Cyclopropane derivatives and their diverse biological activities. Top. Curr. Chem..

[29] Wessjohann L.A., Brandt W., Thiemann T. (2003). Biosynthesis and metabolism of cyclopropane rings in natural compounds. Chem. Rev..

[30] De Meijere A., Kozhushkov S.I., Späth T., Zefirov N.S. (1993). A new general approach to bicyclopropylidenes. J. Org. Chem..

[31] De Meijere A., Kozhushkov S.I., Späth T. (2000). Bicyclopropylidene [Cyclopropane, Cyclopropylidene]. Org. Synth..

[32] Schelper M., de Meijere A. (2005). Facile Construction of Spirocyclopropanated Bi-, Tri- and Tetracyclic Skeletons by Novel Cascades Involving Intra- and Intermolecular HeckReactions of 2-Bromo-1,6-enynes and Bicyclopropylidene. Eur. J. Org. Chem..

[33] Demircan A., Parsons P.J. (1998). Radical addition to substituted furans: Diels-Alder Like products versus Fragmentation Reactions. Synlett.

[34] Demircan A., Parsons P.J. (2003). The Synthesis of Fused Ring Systems Utilising the Intramolecular Addition of Alkenyl Radicals to Furans. Eur. J. Org. Chem..

[35] Karaarslan M., Göktürk E., Demircan A. (2007). Thermal Intramolecular Diels-Alder Reaction of Furan; Synthesis of Nitrogentetracycles, Isobenzofuran And Isobenzothiophene. J. Chem. Res..

[36] Ceylan M., Secen H., Sütbeyaz Y. (1997). Attempted synthesis of cyclopenta-1,2-diene and Wurtz-like condensation products in the reaction of 2,3-dibromocycloalkenes with zinc. J. Chem. Res..

[B37-molecules-19-06058] 37.The diethyl ester corresponding to (7) has previously been prepared Cf. ref. [13]

[38] Kirby A.J. (1980). Effective Molarities for Intramolecular Reactions. Adv. Phys. Org. Chem..

